# Physiological Evaluation of Childcare-Associated Muscle Load on the Neck and Shoulder Region in Japanese Women

**DOI:** 10.1155/2016/1757094

**Published:** 2016-03-15

**Authors:** Saori Yoshinaga, Takuma Kiyokawa, Eriko Kuramoto, Hiroe Kinoshita, Seiji Nemoto

**Affiliations:** ^1^Department of Fundamental Nursing, Faculty of Medicine, Miyazaki University, 5200 Kihara, Kiyotake, Miyazaki 889-1692, Japan; ^2^Graduate School of Nursing Science, Miyazaki University, 5200 Kihara, Kiyotake, Miyazaki 889-1692, Japan; ^3^Department of Humanics Nursing, Miyazaki Prefectural Nursing University, Manabino, Miyazaki 880-0929, Japan; ^4^Interdisciplinary Graduate School of Medicine and Veterinary Medicine, Miyazaki University, 5200 Kihara, Kiyotake, Miyazaki 889-1692, Japan

## Abstract

The awkward movements and postures associated with childcare activities can lead to musculoskeletal symptoms in the neck and shoulders. “Dakko” is a method for carrying a child in Japan, and recently it has been reported to cause shoulder stiffness. To our knowledge, the relationship between childcare activities and the physical load on the neck and shoulders is poorly understood. The present study aims to clarify the muscle load on the neck and shoulder region through dakko simulations. First, the association between dakko movements and trapezius muscle activity is clarified by image and electromyogram analyses. Based on this clarification, the distributions and intensity of the muscle load from repetitive dakko movements are clarified using myogenic potential topography. During dakko movements, trapezius muscle activity was observed when lifting up and setting down the child, but not when holding the child. For the repetitive movements, myogenic electrical potentials were observed in the trapezius region after movement load, and individual characteristics of participants were revealed in both the load distributions and the recovery process. Repetitive dakko movements likely induced sustained muscle tonus in the trapezius, which may be a factor related to shoulder stiffness.

## 1. Introduction

Childcare workers perform a variety of daily tasks, such as carrying, bathing, feeding, and changing diapers that involve many awkward movements and postures. Awkward movements and postures can lead to various musculoskeletal injuries [1, 2]. Unfortunately, many child-rearing behaviors can result in back, neck, and shoulder injuries [3–5].

To improve the quality of health of childcare workers, it is important to disperse the physical load from child-rearing behaviors and prevent symptom aggravation. However, to our knowledge, the relationship between child-rearing behaviors and the physical load on the neck and shoulders is poorly understood.

Electromyogram (EMG) recordings have often been used as objective evaluation indexes of a physical load to understand the characteristics of movement and fatigue [6, 7]. Topography can be used to produce a visual expression of a physiological phenomenon by plotting central values. Image processing is the most useful method for evaluating the amplitude, or localization of huge potentials. We defined myogenic potential topography as an adapting topography that classifies muscle activity according to the intensity of the electrical change generated in the muscle as a color [8]. Elucidating the myogenic potential topography of a neck and the shoulder regions may provide the foundation for visually assessing physical load from child-rearing behaviors.

In Japan, “dakko” is a method of carrying a child, and it has recently been regarded as an important child-rearing behavior that facilitates communication between a parent and child as clinging behavior [9, 10]. However, dakko has been reported to cause shoulder stiffness [11]. Shoulder stiffness is the most common subjective physical discomfort experienced by childcare workers [12, 13].

This study verifies the muscle load on the neck and shoulder region through dakko simulation. First, the association between dakko movements and trapezius muscle activity is clarified by image and EMG analyses. Based on this, the muscle load distribution from repetitive dakko movements is clarified using myogenic potential topography, and the association between dakko as child-rearing behavior and shoulder stiffness is considered.

## 2. Method

### 2.1. Simulations of Dakko

The child to be carried, a 70 cm tall baby model weighing approximately 5.5 kg, was seated in a stroller. The participants were instructed to perform dakko; that is, lift the child from the stroller, hold the child, and then return the child to the stroller, for 1 min.

### 2.2. Participants

**Figure 1:**
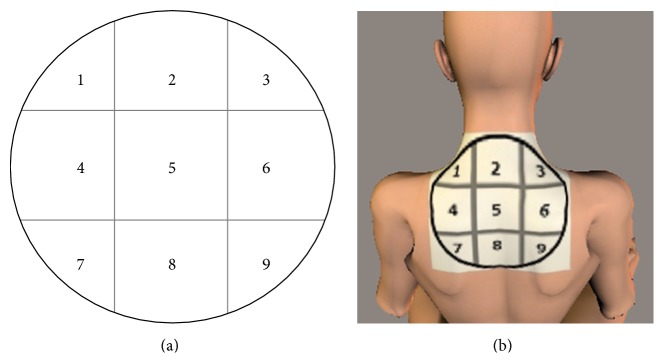
Possible positions of subjective symptoms. (a) The presence of subjective symptoms was investigated at positions numbered 1–9. (b) The mimetic diagram positioned on the back of a body model. The location of subjective symptoms was investigated using the figure classified into the standard six domains: lateral neck regions (1 and 3), medial neck region (2), lateral to the superior scapulae (4 and 6), medial to the superior scapulae (5), lateral to the inferior scapulae (7 and 9), and medial to the superior scapulae (8).

Fifteen healthy, right-handed women with no known neurological or musculoskeletal disorders gave their informed consent to participate in this study. The mean (standard deviation (SD)) physical characteristics were age 26.07 (6.75) yr; height 156.45 (4.75) cm; body weight 49.80 (6.84) kg, and body mass index 20.31 (2.43) kg/m^2^. Each participant filled out a questionnaire regarding the presence of shoulder stiffness (currently experiencing shoulder stiffness, often experiencing shoulder stiffness, or having never experienced shoulder stiffness). Furthermore, the locations of subjective symptoms in terms of stiffness, tension, pressure, and pain, which the participants felt, were assessed using a figure classified into the standard nine domains, as illustrated in Figure 1: lateral neck regions (1 and 3), medial neck region (2), lateral to the superior scapulae (4 and 6), medial to the superior scapulae (5), lateral to the inferior scapulae (7 and 9), and medial to the superior scapulae (8). Participants were permitted to provide multiple answers regarding the location of their body complaint.

This study was approved by the ethics review board of the Department of Medicine at Miyazaki University.

### 2.3. Experimental Protocol and Electromyogram Recordings

First, to clarify the association between the dakko movements and trapezius muscle activity, the dakko movements were video recorded simultaneously with the recording of EMGs. The EMGs were recorded from the middle parts of the trapezius region on the dominant-hand side, using two adhesive surface electrodes (Vitrode L-150X, Nihon Kohden) with an interelectrode distance of 35 mm and a surface electromyography (MEB-900, Nihon Kohden). The reference electrodes were placed over the ear lobes, and the EMG signals were collected bipolarly. The movements of the participants were recorded (HDR-PJ800, Sony) from the dominant-hand side.

**Figure 2:**
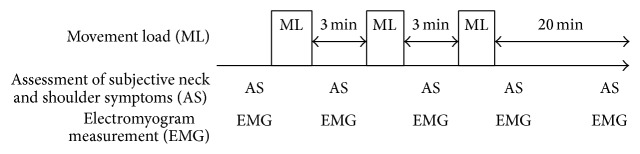
Study protocol.

Second, myogenic potential topography was used to clarify the muscle load distribution due to repetitive dakko movements. Following a 10 min rest, participants performed three sets of the movement load. One set of the movement load comprised 10 repetitions of the dakko movement. For movement load measurements, subjective symptoms of neck and shoulder discomfort were assessed, and EMG data were recorded for 1 min from each participant while relaxed in a sitting posture. As shown in Figure 2, these measurements were repeated before and immediately after the end of the movement load and at the end of convalescence.

**Figure 3:**
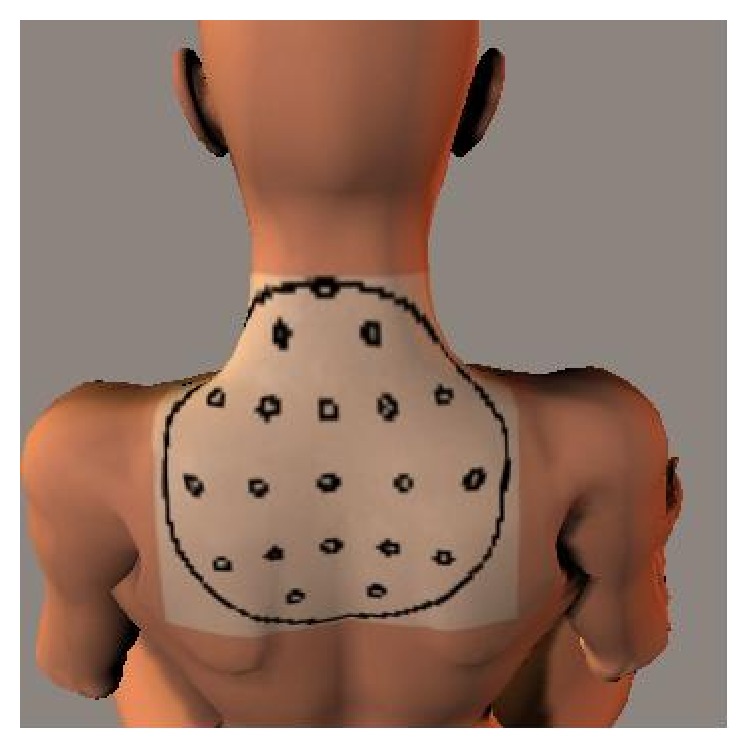
Mimetic diagram showing the electrode arrangement.

Twenty adhesive surface electrodes (Vitrode L-150X, Nihon Kohden), arranged with an interelectrode distance of 35 mm, were used to record the EMGs (EEG-9100, Nihon Kohden). As illustrated in Figure 3, the electrodes were concentrically arranged around the 7th cervical vertebrae in the upper and middle parts of the trapezius after fully removing sebum using alcohol swabs and achieving a skin resistance of 5 kΩ or less. The reference electrodes were placed over the ear lobes, and EMG signals were collected monopolarly. Recording conditions included a time constant of 0.1 s and high-cut filters of 500 Hz. A hum filter was used if needed.

### 2.4. Data Analysis

In the first experiment, muscle activity was analyzed in detail while participants performed the dakko movements. Still images of the recorded dakko movements were extracted every 1 s for each participant. The extracted images were then collated with the EMG wave patterns for the same period, and the association between the movement and amplitude response was examined.

In the second experiment, the EMG data were analyzed using a fast Fourier transform (FFT) to obtain the power spectra. The EEG-9100 (software QP-220A/AK/, Nihon Kohden) was used to create the myogenic potential topography with 6-bit resolution. The maximum potential of the myogenic potential topograms was set at 30 *μ*V^2^ and was subdivided by frequency into five bands: 1–3, 3–5, 5–10, 10–15, and 15–20 Hz. A three-dimensional female body model was made using a computer-character design tool (POSER4, e-frontier, Inc.), and the myogenic potential topograms were mapped onto the model as a texture.

## 3. Results

### 3.1. Characteristics of the Participants

According to the questionnaire results, eight participants were classified as having shoulder stiffness, while seven participants were classified as not having shoulder stiffness. For the repetitive dakko movements, participants A to H reported experiencing stiffness, pain, pressure, and tension centering on their neck and scapular region that were present prior to movement load and that continued until the end of convalescence. While participants I to O reported not experiencing shoulder stiffness normally, they noted stiffness, pain, pressure, and tension centering on the neck and scapular region immediately after the end of movement load, which continued until the end of convalescence for some participants (Table 1).

### 3.2. Muscle Activity of the Neck and Shoulder Region from Dakko Movements

Images of participants lifting the child showed that 14 participants supported the child vertically, and one participant supported the child horizontally. Regarding the posture of the lifting movement, 14 participants flexed both lower legs and bent forward. One participant did not flex both lower legs but bent forward. When holding the child, 14 participants held the child vertically with both arms and then supported the buttocks of the child with their dominant-hand. One participant held the child horizontally, supported the upper part of the body of the child using her nondominant arm, and supported the buttocks of the child with her dominant-hand. When returning the child to the stroller, 14 participants supported the child vertically, and one participant supported the child horizontally.

**Figure 4:**
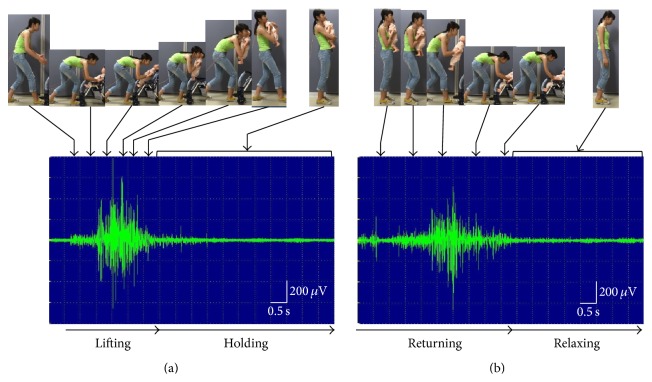
Photographs and electromyograms of the “dakko” movement from participant N. (a) An increase in the EMG amplitude was observed when lifting the child from the stroller. When holding the child, changes in the amplitude and frequency were not observed. (b) An increase in the EMG amplitude was observed when returning the child to the stroller.

Regarding the amplitude response corresponding to these images, during lifting, the EMG amplitude began to increase when the hand of the participant first touched the child and reached a maximum as the child was brought close to the trunk of the participant. When holding the child, no changes in the EMG amplitude were observed. When returning the child to the stroller, the EMG amplitude began to increase gradually as the child was moved away from the trunk of the participant and reached a maximum just before the child was placed in the stroller. A similar tendency in the amplitude response was observed for all participants.  Figure 4 shows a representative case.

### 3.3. Chronological Change of Muscle Load from Repetitive Dakko Movements

**Figure 5:**
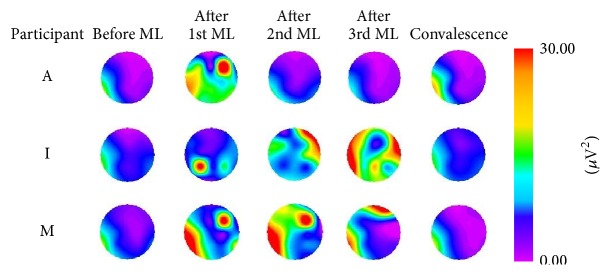
Myogenic potential topograms of participants A, I, and M. After movement load (ML), the change in high potential mainly appeared in the 1–3 Hz frequency band but disappeared by the end of convalescence.

**Figure 6:**
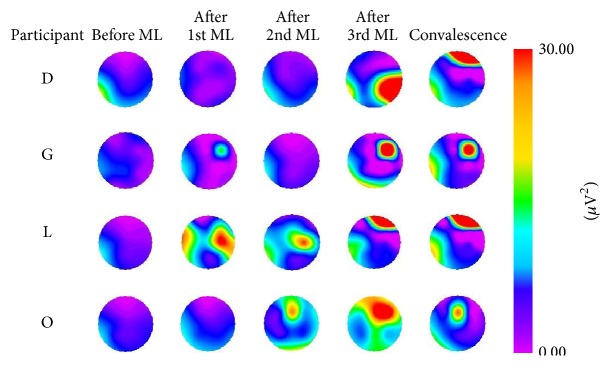
Myogenic potential topograms of participants D, G, L, and O. After movement load (ML), the change in high potential mainly appeared in the 1–3 Hz frequency band, and the high potential was still observed at the end of convalescence.

**Figure 7:**
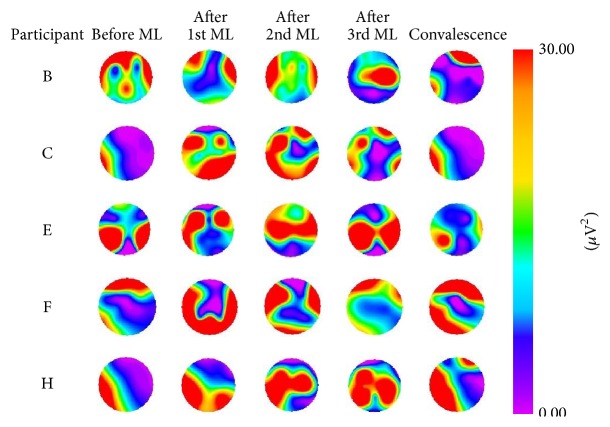
Myogenic potential topograms of participants B, C, E, F, and H. Before movement load (ML), the high potential mainly appeared in the 1–3 Hz frequency band, and the high potential was still observed at the end of convalescence.

**Figure 8:**
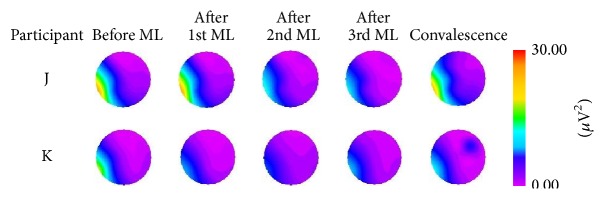
Myogenic potential topograms of participants J and K. No remarkable change in potential was observed before or after the movement load (ML) or at the end of convalescence in the 1–3 Hz frequency band.

By repeating the dakko movements, changes in color representing electrical activity after the movement load were observed on myogenic potential topograms. Topograms were classified into four patterns based on the appearance of the electrical activity. For pattern 1, after the 1st movement load, participants A, I, and M exhibited a change of relatively high potential (>30 *μ*V^2^) in the low-frequency (1–3 Hz) domain of the EMG, as illustrated in Figure 5. The high potential distribution had disappeared by the end of convalescence. For pattern 2, after the 1st, 2nd, or 3rd movement load, participants D, G, L, and O exhibited a change of relatively high potential (>30 *μ*V^2^) in the low-frequency (1–3 Hz) domain of the EMG. The high potential distribution was still observed at the end of convalescence, as illustrated in Figure 6. For pattern 3, participants B, C, E, F, and H exhibited a change of relatively high potential (>30 *μ*V^2^) in the low-frequency (1–3 Hz) domain of the EMG before the movement load. The high potential distribution was still observed at the end of convalescence, as illustrated in Figure 7. For pattern 4, no remarkable changes in potential were observed before or after the movement load or at the end of convalescence in participants J and K, as illustrated in Figure 8.

**Figure 9:**
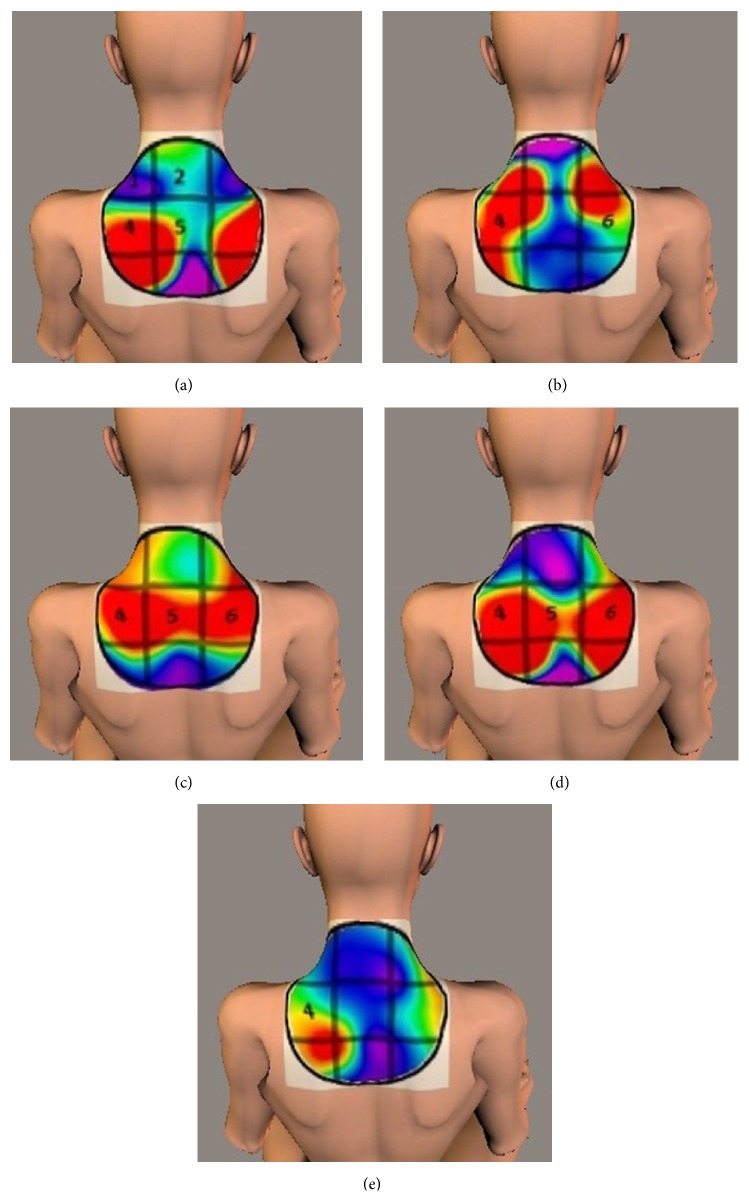
Projection of myogenic potential topograms from participant E onto a three-dimensional female body model. The numbers represent the locations of subjective symptoms. (a) (Before ML) changes in electrical activity were located lateral to the superior scapular regions. Subjective symptoms were located mainly on the left side. (b) (After 1st ML) changes in electrical activity were located on both sides of the neck and near the superior scapulae. The locations of subjective symptoms resembled areas exhibiting changes in the electric potential. (c) (After 2nd ML) changes in electrical activity were located medial to the superior scapulae. Subjective symptoms originated in areas exhibiting changes in the electric potential. (d) (After 3rd ML) similar to (c), changes in electrical activity were located medial to the superior scapulae, and subjective symptoms originated in areas exhibiting changes in the electric potential. (e) (Convalescence) changes in electrical activity were located lateral to the inferior scapulae. The locations of subjective symptoms resembled the area exhibiting a change in the electric potential.

When the myogenic potential topograms were mapped onto the model, the high potential was distributed over the middle and upper parts of the trapezius, proximal to the neck and scapular regions. Comparison of the myogenic potential topography with subjective symptoms confirmed that the distribution of myogenic potentials corresponded to the location of the subjective symptoms.  Figure 9 shows a representative case.

## 4. Discussion

This is the first study to clarify objectively the load on the neck and shoulder region from dakko movements. First, this study verified the association between dakko movements and trapezius muscle activity by image and EMG analyses. Although one participant used a different posture when lifting up, holding, and setting down the child, the EMG wave patterns of all participants exhibited a similar tendency. The EMG amplitude increased as the child was lifted from and returned to the stroller. This increase in amplitude indicated that a greater number of action potentials occurred during these parts of the dakko movement; that is, there was a greater number of contracting muscle fibers. Thus, it was confirmed that dakko movements require trapezius muscle activation when the child is lifted from and returned to the stroller. When holding the child, remarkable muscle activity of the middle parts of the trapezius was not observed in any of the participants, indicating that the middle parts of the trapezius was not directly activated when holding the child.

The trapezius muscle is of special interest in studies on the pathogenesis of shoulder stiffness [14, 15]. In occupational and laboratory settings, EMG recordings have often been used to gain insight into the activity of the trapezius muscle. For instance, Itoh et al. [16] identified the electrical activities that correspond with the sensation of dull shoulder pain among patients with shoulder stiffness. Additionally, Leonard et al. [17] reported that the mean EMG activity of the upper trapezius muscle was significantly higher in subjects with neck pain than in those without. Results from these earlier studies indicate that shoulder stiffness may be evident in the characteristics of trapezius muscle activity.

Childcare workers repeat dakko movements many times daily [11]. Therefore, this study examined the association between dakko movements and shoulder stiffness by evaluating the muscle activity of the trapezius regions during repetition of the movements. Activity of the trapezius before and immediately after movement load and at the end of convalescence was observed, and myogenic potential topography was used to identify visually the distribution and intensity of the muscle activity.

By repeating the movement load, participants exhibited remarkable electrical change in the low-frequency domain (1–3 Hz) of EMGs after movement load. The strength of a muscle contraction depends on the discharge frequency and mobilization of alpha motor neurons. Mobilization of a motor unit mainly takes the lead during a weak contraction, and the discharge frequency of alpha motor neurons greatly increases as the contraction intensifies. Moreover, frequency analysis indicates that an increase in contraction intensity reflects an increase in frequency [18, 19]. Since the increase in potential in this research was remarkable in the 1–3 Hz low-frequency bands, it was surmised to represent muscle activity related to weak muscle contraction. Therefore, it was thought that involuntary contractions induced in the trapezius of participants after movement load sustained muscle tonus. This sustained muscle contraction causes muscle rigidity and ischemia, which are factors related to shoulder stiffness. Thus, the high potential changes after the movement load may be a sign of shoulder stiffness due to the muscle load associated with dakko.

Moreover, by projecting myogenic potential topograms on a body model, high potential regions were found to be distributed not throughout the entire trapezius but only in limited areas of the neck or scapular region. Furthermore, when the relevance between a myogenic potential topogram and actual complaint was examined, the distribution patterns of the myogenic potential and subjective symptoms were in agreement. Some previous studies revealed the presence of muscular activity as a nociceptive response to an algesic substance [20–22]. A high concentration of serotonin and glutamate in the interstitial fluid surrounding the trapezius muscle has also been reported in a subject with shoulder stiffness [23]. Although many factors can induce involuntary muscle contractions, involuntary contractions induced in the trapezius in study participants may be a nociceptive response to repetitive movements.

Further, chronological changes in muscle load from repetitive dakko movements were classified into four patterns based on the appearance of the high electrical potential. For pattern 1, the high potential appeared after the 1st movement load and disappeared by the end of convalescence. For pattern 2, the high potential appeared after the 1st, 2nd, or 3rd movement load and was still present at the end of convalescence. For pattern 3, the high potential appeared before the movement load and was still present at the end of convalescence. For pattern 4, no remarkable changes in potential were observed before or after movement load or at the end of convalescence. Regarding the participants classified as exhibiting patterns 2 and 3, the sustained muscle tonus with movement load continued during convalescence. These participants lagged behind in recovery of the muscle tonus compared to participants classified as exhibiting patterns 1 and 4, and this delay in recovery likely contributes to the accumulation of load on the muscles of the neck and shoulders. Moreover, high myogenic potential changes before the movement load were seen in five participants classified as exhibiting pattern 3. These participants, classified as having shoulder stiffness, noticed symptoms in the neck and shoulder region before repetition of the movement and were suspected of having routine strain on the trapezius. Therefore, the repetitive dakko movements were a factor in the delayed recovery of the muscle tonus and exacerbated shoulder stiffness. Moreover, these results show that the high electrical potential distributions and process of recovering from sustained muscle tonus due to repetitive dakko movements have individual characteristics. Because the physical response to the movement load varied between participants, personalized interventions to alleviate shoulder stiffness may be possible by understanding the individual characteristics of the muscle load.

## 5. Conclusions

For dakko movements, trapezius muscle activity was observed when lifting up and setting down a child, but not when holding a child. Moreover, from repetition of these movements, participants exhibited remarkable electrical changes in the low-frequency domain (1–3 Hz) of EMGs after the movement load. For individual participants, the high electrical potential differed in both the distributions and the recovery process. This study suggests that dakko movements cause sustained muscle tonus in childcare workers, which is a factor related to shoulder stiffness.

## Figures and Tables

**Table 1:** Characteristics of participants.

Participants	Subjective symptoms (position number)				
	Before ML	After 1st ML	After 2nd ML	After 3rd ML	Convalescence
A (SS)	None	Tension (5)	Pain (4, 5, 6)	Pressure (4, 5, 6)	Tension (4, 5, 6)
B (SS)	Tension (all areas)	Pressure (6)	Pressure (6)	Tension (5, 6)	Tension (all areas)
C (SS)	Pressure (4, 5, 6)	Pressure (4, 5, 6)	Stiffness (4, 5, 6)	Stiffness (4, 5, 6)	Stiffness (4, 5, 6)
D (SS)	None	Tension (1, 2, 3)	Tension (1, 2, 3)	Pressure (2, 3, 6)	None
E (SS)	Stiffness (1, 2, 4, 5)	Stiffness (4, 6)	Pain (4, 5, 6)	Pressure (4, 5, 6)	Stiffness (4)
F (SS)	Pressure (1, 2, 4, 5)	Pressure (1, 2, 4, 5)	Pressure (1, 2, 4, 5)	Pressure (2, 5, 6)	Pressure (4, 5)
G (SS)	Stiffness (5)	Stiffness (5)	Tension (4, 5)	Tension (4, 5)	Tension (5)
H (SS)	None	Pressure (4, 6)	Pressure (1, 2, 3, 4, 6)	Pressure (1, 2, 3, 4, 6)	Tension (1, 2, 3)
I (NSS)	None	None	None	None	None
J (NSS)	None	Stiffness (2, 3)	Stiffness (2, 3, 5)	Stiffness (2, 3, 5)	Stiffness (2, 3)
K (NSS)	None	None	None	None	None
L (NSS)	None	Stiffness (5, 6)	Pain (2, 3, 5, 6)	Pain (5)	Stiffness (5, 6)
M (NSS)	None	Pressure (4, 5)	Pressure (4, 5)	Pressure (1, 2, 3, 5)	None
N (NSS)	None	None	None	None	None
O (NSS)	None	None	Tension (5)	None	None

SS: shoulder stiffness, NSS: no shoulder stiffness, and ML: movement load.
